# DNA methylation signature of interleukin 1 receptor type II in asthma

**DOI:** 10.1186/s13148-015-0114-0

**Published:** 2015-08-05

**Authors:** Valérie Gagné-Ouellet, Simon-Pierre Guay, Anne-Marie Boucher-Lafleur, Luigi Bouchard, Catherine Laprise

**Affiliations:** Département des sciences fondamentales, Université du Québec à Chicoutimi, Chicoutimi, QC Canada; Department of Biochemistry, Université de Sherbrooke, Sherbrooke, QC Canada; ECOGENE-21 and Lipid Clinic, Hôpital de Chicoutimi, Saguenay, QC Canada

**Keywords:** Epigenetics, Methylation, IL1, IL1R1, IL1R2, Asthma, Atopy

## Abstract

Interleukin 1 and its receptors are associated with allergic diseases such as asthma. In the present study, we measured DNA methylation at the *IL1R1* and *IL1R2* gene loci and assessed for associations with asthma-related phenotypes and gene expressions. We found that asthmatic and atopic individuals have higher *IL1R2* promoter DNA methylation than control subjects. Additionally, we observed a negative correlation between DNA methylation at the *IL1R2* promoter and *IL1R2* mRNA expression. These results suggest for the first time that *IL1R2* promoter DNA methylation is associated with its gene repression in allergic diseases such as asthma.

**Electronic supplementary material:**

The online version of this article (doi:10.1186/s13148-015-0114-0) contains supplementary material, which is available to authorized users.

## Introduction

Interleukin 1 (IL1) plays a key role in the inflammatory process of asthma [1]. We reported the association of polymorphisms within the IL1 receptors type I (*IL1R1*) and type II (*IL1R2*) gene loci with asthma and atopy in the French Canadian Saguenay–Lac-Saint-Jean (SLSJ) asthma study [2, 3]. The *IL1R2* gene expression signature in allergic asthma has also been described [4–6]. Epigenetics has received tremendous attention, and variations in DNA methylation (DNA-Me) in candidate genes have been reported associated with asthma and allergic related disorders [7–12]. These findings underline the relevance of genetic and epigenetic profiling to identify pathways associated with allergic diseases. Such a combined approach will facilitate the understanding of the functional impacts of genetic and epigenetic variations on transcription and molecular mechanisms involved in allergic diseases. In this study, we hypothesized that DNA-Me in the promoters of *IL1R1* and *IL1R2* is associated with asthma and/or atopy.

## Patients and methods

**Fig. 1:**
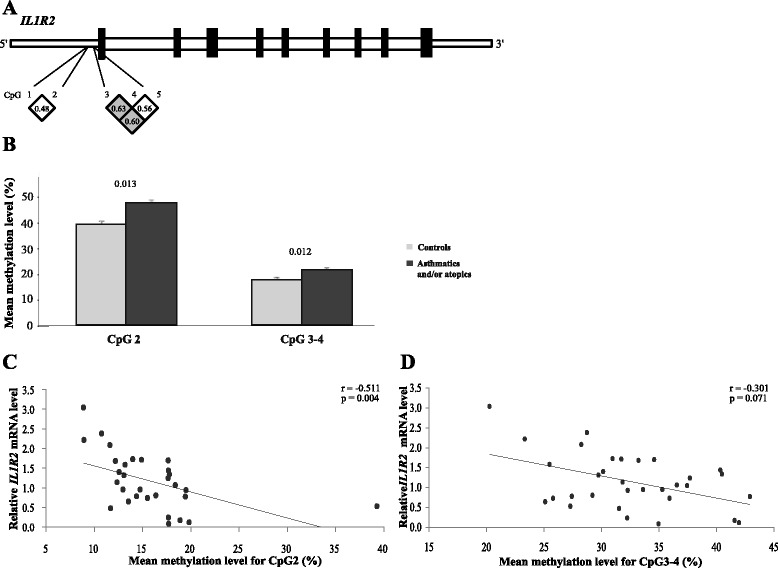
Association between CpGs’ DNA methylation levels for *IL1R2* and gene expression in asthma and atopy. **a** Schematic representation of *IL1R2*, location of epigenotyped CpG sites, and pairwise correlations between CpG sites. **b** Mean DNA-Me levels for CpG2 and CpG3-4 of *IL1R2* in control and affected subjects (individuals with asthma, atopy, or both). **c** Correlation between DNA-Me level of *IL1R2*-CpG2 and mRNA level. **d** Correlation between mean DNA-Me level of *IL1R2-*CpG3 and 4 and mRNA level

Clinical characteristics of the 93 individuals (21 non-atopic asthmatic, 26 atopic asthmatic and 21 atopic individuals, and 25 non-asthmatic non-atopic controls) from the Saguenay–Lac-Saint-Jean asthma familial collection [13] and included in the analysis are shown in Table 1. Ethics committee approved the study, and all subjects gave informed consent. Based on previous genetic [14] and epigenetic analyses [15], methylation at 1 CpG in promoter and 3 CpGs in exon 1 of *IL1R1* (Additional file 1: Figure S1) and 5 CpGs in promoter of *IL1R2* (Fig. 1a) was measured. DNA-Me differences (Δ*β*) between affected (individuals with asthma, atopy, or both) and non-affected individuals were assessed and DNA-Me was correlated with gene expression for each CpG. DNA-Me was measured on DNA extracted from blood (blood and cell culture Midi kit, Qiagen, Canada) using bis-pyrosequencing (EpiTech Bisulfite Kits, Pyromark PCR Kit, Pyromark Gold Q24 Reagents, Qiagen, Canada). PCR primers were designed using PyroMark Assay Design software (v2.0.1.15). Total RNA was extracted from whole blood (RNeasy Plus Mini Kit, Qiagen, Canada) using a subset of affected and non-affected individuals (*n* = 30). For each sample, RNA was converted into cDNA (qScript™ cDNA SuperMix, Quanta Biosciences, USA), and mRNA quantification was determined (PerfeCTa® qPCR FastMix®, Quanta Biosciences, USA) using the two standard curves method with *RPLP0* as a reference gene [16].

**Table 1:** Clinical characteristics of individuals from the Saguenay―Lac-Saint-Jean asthma familial collection

Characteristics	All individuals (*n* = 93)	Controls (*n* = 25)	Asthmatics^a^ and/or atopics^b^ (*n* = 68)
Sex ratio (M:F)	1:1.2	1:1.8	1:1
Mean age, year (range)	15 (3–46)	14 (3–44)	15 (4–46)
<16 years old, *n* (%)	64 (69)	18 (72)	46 (68)
FEV_1_, % predicted (SD)^c^	62 (40)	63 (40)	62 (40)
PC_20_, mg/ml (SD)^d^	8.2 (4.3)	15.3 (3.4)	6.8 (4.4)
Serum IgE, μg/l (SD)^e^	109 (5)	36 (4)	157 (4)
Asthma, *n* (%)^a^	47 (51)	NA	47 (69)
Atopy, *n* (%)^b^	47 (51)	NA	47 (69)
With asthma, *n* (%)^a^	26 (28)	NA	26 (38)

^a^Present asthma or past documented clinical history of asthma. Data available for all individuals

^b^Defined as having at least one positive response on the skin prick test (wheal diameter ≥3 mm at 10 min). Data available for all individuals

^c^FEV_1_ = mean and standard deviation (SD) calculated for forced expiratory volume in 1 s for 67 individuals (16 controls, 51 asthmatic and/or atopic individuals)

^d^PC_20_ = geometric mean and SD of provocative methacholine concentration inducing 20 % decline in FEV_1_ calculated for 58 individuals (14 controls, 44 asthmatic and/or atopic individuals)

^e^IgE = geometric mean and SD of serum immunoglobulin (Ig) E level concentration calculated for 80 individuals (20 controls, 60 asthmatic and/or atopic individuals)

The association between *IL1R1* and *IL1R2* DNA-Me levels and asthma and/or atopy at each CpG was analyzed by logistic regression considering age and sex as covariates [9]. Gene expression analysis by phenotype was not performed as control group sample size was insufficient (*n* = 4). The association between DNA-Me and mRNA levels was assessed by Spearman correlation. CpG dinucleotides with *r* > 0.6 were combined before they were tested for associations with asthma and/or atopy and for correlation with gene expressions. Δ*β* with *p* value < 0.05 was considered statistically significant. Statistical analyses were conducted using the statistical software SPSS (v11.5.0, USA).

## Results

In this study, we detected higher levels of DNA-Me at *IL1R2* among affected individuals (i.e., with asthma, atopy, or both) as compared to non-affected controls (Δ*β* = 8.02 %, *p* value *=* 0.013, and Δ*β* = 3.72 %, *p* value = 0.012 for *IL1R2*-CpG2 and the mean for CpG3 and CpG4, respectively (Table 2, Fig. 1b)). Atopic and non-atopic asthma were associated with DNA-Me at *IL1R2* but not atopy alone (data not shown). We also observed that DNA-Me at *IL1R2*-CpG2 was negatively correlated with its mRNA levels (*r* = −0.511, *p* value = 0.004) (Fig. 1c), but it was not correlated for CpG3 and CpG4 (Fig. 1d).

**Table 2:** Summary of DNA methylation analysis on promoter of two interleukin 1 receptors in whole blood samples from Saguenay―Lac-Saint-Jean asthma familial collection

Gene	CpG	Δ*β* ^a^	*p* value
*IL1R1* promoter and exon 1	1	1.19	0.113
	2–4	0.22	0.892
*IL1R2* promoter	1	−0.59	0.569
	2	8.02	*0.013*
	3–4	3.72	*0.012*
	5	−0.50	0.564

Significant *p* values are shown in italics

^a^Δ*β* are calculated with mean methylation ratio for asthmatic and/or atopic individuals on control individuals

## Discussion

An epigenetic signature has also been identified for *IL1R2* promoter in systemic lupus erythematosus (SLE) [15]. The risk of allergic disorders was significantly increased in SLE patients, which suggests that these conditions share some common biomarkers [17]. The negative correlation we observed between DNA-Me and gene expression levels for *IL1R2* may be due to stoichiometry. Methylation may limit access of a transcription factor to DNA and hinders transcriptions [18]. We identified potential binding sites for transcription factors relevant to asthma near the CpG dinucleotide sites of *IL1R2* analyzed (Additional file 2: Figure S2) which could explain the inverse correlation between methylation and gene expression [19]. Noteworthy is the potential binding site for nuclear factor kappa B/c-rel (NFKB) at the *IL1R2* promoter; it is involved in inflammation through several pathways, including IL1 signalization [20]. Given that IL1R2 acts as a decoy receptor to antagonize the bound ligand [21], our data prompted the speculation that hypermethylation of *IL1R2* in asthma and atopy negatively regulates *IL1R2* expression and less decoy receptors are available to reduce the downstream pro-inflammatory response of IL1 in the presence of unchanged IL1R1 level [22, 23]. Unlike IL1R1, IL1R2 does not have an intracellular domain and the formation of IL1-IL1R2 complex inactivates the IL1 downstream signaling cascade; hence, silences the role of IL1 in inflammation. Functional study will be needed to investigate the impact of observed epi-variations on the production of expressed receptors. This hypothesis could be attributed to both asthma and atopy as IL1R2 non-signaling receptor is suspected to influence Th2 imbalance [24], and both disorders are driven by Th2 allergic lung inflammation [25, 26].

To our knowledge, this is the first report of (1) a hypermethylation signature of *IL1R2* promoter in asthma with or without atopy and (2) an inverse correlation between methylation at *IL1R2* promoter and its gene expression. Together, they underline the relevance of IL1R2 as a potential biomarker of asthma and atopy. Further work is needed to understand the interactions between environmental exposures and epigenetic modifications like the ones identified in this study. Such understanding will aid the discovery of disease mechanisms associated and development of more effective therapies.

## Additional files

Additional file 1: Figure S1. Schematic representation of IL1R1 and location of epigenotyped CpG sites. This figure illustrates a simplified schematic representation of *IL1R1* and location of selected CpG dinucleotide sites and pairwise correlations between each CpG. Additional file 2: Figure S2. Potential binding sites for transcription factors inIL1R1and IL1R2. This figure shows a segment of the primary sequences of *IL1R1* and *IL1R2*. Both sequences lie in the respective gene promoter (UCSC Genome Browser assembly GRCh38/hg38). Epigenotyped CpG sites are shown in blue and are numbered from the distal part of the promoter. Exons are shown in red. Potential binding sites for transcription factors in differentially methylated loci are shown by underlined sequences, and the name of the transcription factors are indicated underneath.

## Abbreviations

CpG: cytosine-phosphate-guanine
DNA-Me: DNA methylation
IL1: interleukin 1
IL1R1: interleukin 1 receptor type 1
IL1R2: interleukin 1 receptor type 2
RPLP0: ribosomal protein, large, P0
SLE: systemic lupus erythematosus
SLSJ: Saguenay–Lac-Saint-Jean
Δ*β*: difference of methylation

## References

[1] Dinarello CA (1996). Biologic basis for interleukin-1 in disease. Blood.

[2] Daley D, Lemire M, Akhabir L, Chan-Yeung M, He JQ, McDonald T, Sandford A, Stefanowicz D, Tripp B, Zamar D (2009). Analyses of associations with asthma in four asthma population samples from Canada and Australia. Hum Genet.

[3] Daley D, Park JE, He JQ, Yan J, Akhabir L, Stefanowicz D, Becker AB, Chan-Yeung M, Bosse Y, Kozyrskyj AL (2012). Associations and interactions of genetic polymorphisms in innate immunity genes with early viral infections and susceptibility to asthma and asthma-related phenotypes. J Allergy Clin Immunol.

[4] Laprise C, Sladek R, Ponton A, Bernier MC, Hudson TJ, Laviolette M (2004). Functional classes of bronchial mucosa genes that are differentially expressed in asthma. BMC Genomics.

[5] Chamberland A, Madore A-M, Tremblay K, Laviolette M, Laprise C (2009). A comparison of two sets of microarray experiments to define allergic asthma expression pattern. Exp Lung Res.

[6] Pociot F, Molvig J, Wogensen L, Worsaae H, Nerup J (1992). A TaqI polymorphism in the human interleukin-1 beta (IL-1 beta) gene correlates with IL-1 beta secretion in vitro. Eur J Clin Invest.

[7] Morales E, Bustamante M, Vilahur N, Escaramis G, Montfort M, de Cid R, Garcia-Esteban R, Torrent M, Estivill X, Grimalt JO (2012). DNA hypomethylation at ALOX12 is associated with persistent wheezing in childhood. Am J Respir Crit Care Med.

[8] Reinius LE, Gref A, Saaf A, Acevedo N, Joerink M, Kupczyk M, D’Amato M, Bergstrom A, Melen E, Scheynius A (2013). DNA methylation in the neuropeptide S receptor 1 (NPSR1) promoter in relation to asthma and environmental factors. PLoS One.

[9] Naumova AK, Al Tuwaijri A, Morin A, Vaillancourt VT, Madore AM, Berlivet S, Kohan-Ghadr HR, Moussette S, Laprise C (2013). Sex- and age-dependent DNA methylation at the 17q12-q21 locus associated with childhood asthma. Hum Genet.

[10] Wang IJ, Karmaus WJ, Chen SL, Holloway JW, Ewart S (2015). Effects of phthalate exposure on asthma may be mediated through alterations in DNA methylation. Clin Epigenetics.

[11] Seumois G, Chavez L, Gerasimova A, Lienhard M, Omran N, Kalinke L, Vedanayagam M, Ganesan AP, Chawla A, Djukanovic R (2014). Epigenomic analysis of primary human T cells reveals enhancers associated with TH2 memory cell differentiation and asthma susceptibility. Nat Immunol.

[12] Yang IV, Pedersen BS, Liu A, O’Connor GT, Teach SJ, Kattan M, Misiak RT, Gruchalla R, Steinbach SF, Szefler SJ (2015). DNA methylation and childhood asthma in the inner city. J Allergy Clin Immunol.

[13] Laprise C (2014). The Saguenay-Lac-Saint-Jean asthma familial collection: the genetics of asthma in a young founder population. Genes Immun.

[14] Smith AJ, Keen LJ, Billingham MJ, Perry MJ, Elson CJ, Kirwan JR, Sims JE, Doherty M, Spector TD, Bidwell JL (2004). Extended haplotypes and linkage disequilibrium in the IL1R1-IL1A-IL1B-IL1RN gene cluster: association with knee osteoarthritis. Genes Immun.

[15] Lin SY, Hsieh SC, Lin YC, Lee CN, Tsai MH, Lai LC, Chuang EY, Chen PC, Hung CC, Chen LY (2012). A whole genome methylation analysis of systemic lupus erythematosus: hypomethylation of the IL10 and IL1R2 promoters is associated with disease activity. Genes Immun.

[16] Wang T, Liang ZA, Sandford AJ, Xiong XY, Yang YY, Ji YL, He JQ (2012). Selection of suitable housekeeping genes for real-time quantitative PCR in CD4(+) lymphocytes from asthmatics with or without depression. PLoS One.

[17] Shen TC, Tu CY, Lin CL, Wei CC, Li YF (2014). Increased risk of asthma in patients with systemic lupus erythematosus. Am J Respir Crit Care Med.

[18] Blattler A, Farnham PJ (2013). Cross-talk between site-specific transcription factors and DNA methylation states. J Biological Chemistry.

[19] Turker MS (2002). Gene silencing in mammalian cells and the spread of DNA methylation. Oncogene.

[20] Acuner Ozbabacan SE, Gursoy A, Nussinov R, Keskin O (2014). The structural pathway of interleukin 1 (IL-1) initiated signaling reveals mechanisms of oncogenic mutations and SNPs in inflammation and cancer. PLoS Comput Biol.

[21] Colotta F, Re F, Muzio M, Bertini R, Polentarutti N, Sironi M, Giri JG, Dower SK, Sims JE, Mantovani A (1993). Interleukin-1 type II receptor: a decoy target for IL-1 that is regulated by IL-4. Science.

[22] Colotta F, Dower SK, Sims JE, Mantovani A (1994). The type II ‘decoy’ receptor: a novel regulatory pathway for interleukin 1. Immunol Today.

[23] Garlanda C, Dinarello CA, Mantovani A (2013). The interleukin-1 family: back to the future. Immunity.

[24] Sims JE, Gayle MA, Slack JL, Alderson MR, Bird TA, Giri JG, Colotta F, Re F, Mantovani A, Shanebeck K (1993). Interleukin 1 signaling occurs exclusively via the type I receptor. Proc Natl Acad Sci U S A.

[25] Islam SA, Luster AD (2012). T cell homing to epithelial barriers in allergic disease. Nat Med.

[26] Paul WE. History of interleukin-4. Cytokine 2015. 10.1016/j.cyto.2015.01.038. 10.1016/j.cyto.2015.01.038 PMC4532601 25814340

