# Supplementary figures and images for: DNA methylation signature of interleukin 1 receptor type II in asthma
Source: Clin Epigenetics. 2015 Aug 5;7(1):80. doi: 10.1186/s13148-015-0114-0 (PMC4526162; doi:10.1186/s13148-015-0114-0)

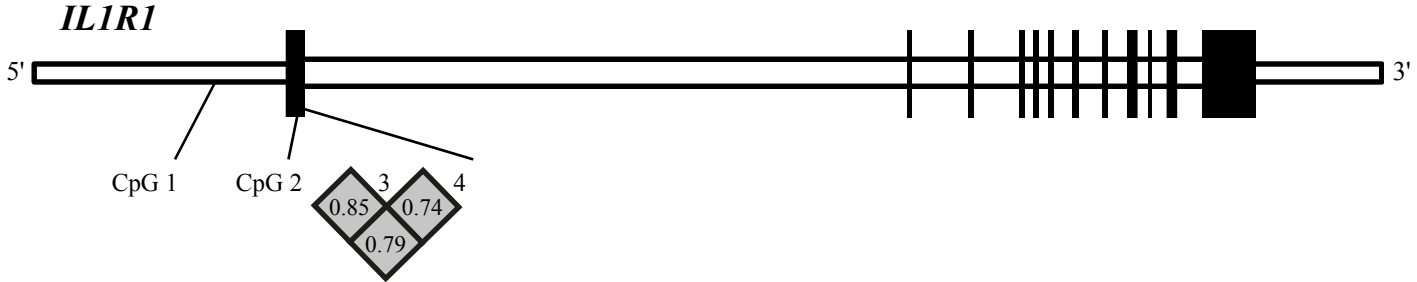

Supplement: Additional file 1: Figure S1. — Schematic representation of IL1R1 and location of epigenotyped CpG sites. This figure illustrates a simplified schematic representation of IL1R1 and location of selected CpG dinucleotide sites and pairwise correlations between each CpG. [file 13148_2015_114_MOESM1_ESM.pdf]
