# Supplementary material for: DNA methylation signature of interleukin 1 receptor type II in asthma
Source: Clin Epigenetics. 2015 Aug 5;7(1):80. doi: 10.1186/s13148-015-0114-0 (PMC4526162; doi:10.1186/s13148-015-0114-0)
Supplement: Additional file 2: Figure S2. — Potential binding sites for transcription factors inIL1R1and IL1R2. This figure shows a segment of the primary sequences of IL1R1 and IL1R2. Both sequences lie in the respective gene promoter (UCSC Genome Browser assembly GRCh38/hg38). Epigenotyped CpG sites are shown in blue and are numbered from the distal part of the promoter. Exons are shown in red. Potential binding sites for transcription factors in differentially methylated loci are shown by underlined sequences, and the name of the transcription factors are indicated underneath. [file 13148_2015_114_MOESM2_ESM.pdf]

IL1R1 - CpG1-4

CCCTCCAGCTCACAGGTATCTGGCCAGGGTGGGGGCACCGCTGTGTTGTAGAC

ATGCTCCTTAAAGAGTGCTTTGTTCCCACTTGGAGTAGGGGACCCTCCCTCATA

GGGCTGTGGCCTCTCTGCCAGCCAGGAAGGGCCTGAAGTCTGATGGAGGAGG

1

GCATCTGAGGACAGGGTCTGAGAGTACACGGTGTGCTGGCCTGCTCTGTGGCT

GREF

GGGGTCCTTGGGCGACTGCAGGCTGAGGCTGCTCAGCAGTGAGGAATGCTGA

GTCAAGAGGATGAACAGCTTCTTTCTGAGAAGCCAGGGTGATCGCTGAGTCAT

GCCACTCTTGGCTAACACATGTGCAGCCTGCCCGGGGCTTTAGGGCTGAGCAT

AACCAGTCCTGCTTTTTATCTTCTCTTTCTCAACCCCATCTCTTCTTCAGGAAAC

2 3

ATTTACCAGATGTGTGGCTTGAGTGTCCATTCCCGCAACAAAAACGCTCTGCAG

4

TAAAGCTCCGAGATGGCTGTGTTTGTGTTGGACTGCTCTAGATG

IL1R2 - CpG1-5

TAACAGTTAAAAATCATACAGGACTCTGAAAACAAAACAAAACAAAAACTT

AACCTTTCTTAGATCCTAAAAGCGTTCCTTGGATGGAAAACCAACTCTTCCAC

TGGCTAAAGATCAAAAGCATCTTTTTCTCTTCAATGGGGCCACAGTTGGGCAA

1 2 3

AACGCCCATCACTTTAAAACCACCTCTCGGCTGGAAGTACGTAATTTTTAGCG

ETS1

4 5

AGTCACAGAAAAATAGGGAAACTTATGCGGGCGTTTCCTTGGCCACTTCCCCA

NFKB XBBF

TCTGGGTGATCATGTACTCAGACCCAGCACTGCAGCCTGGGGGTGCTCCCCG

TGAGGAGGAAAAGGTGTGTCCGCTGCCACCCAGTGTGAGCAGGTGACACCAC
